# Controlled Maxillofacial Administration of Levodopa in a Patient With Parkinson’s Disease: A First-in-Human 24-Hour Follow-Up Study

**DOI:** 10.7759/cureus.107436

**Published:** 2026-04-21

**Authors:** Suresh C Thirunavukarasu, B B V Ramanan, Devi Varadharaj, Durairaj Arumugam, Jayasri Poyyadhappan, Deepika Moorthy, Simulia Dhinju Benjamin, Kavita Verma, Jishnu Sudhakar, Hridwik Adiyeri Janardhanan, Anoop UR

**Affiliations:** 1 Neurology, Indira Gandhi Government General Hospital and Post Graduate Institute, Puducherry, IND; 2 General Medicine, Swamy Vivekananda Medical College Hospital and Research Institute, Tiruchengode, IND; 3 General Medicine, Indira Gandhi Medical College and Research Institute, Puducherry, IND; 4 Neurological Physiotherapy, Jijamata College of Physiotherapy, Majalgaon, IND; 5 Research and Development, UR Anoop Research Group, Puducherry, IND

**Keywords:** area under the concentration-time curve, high-performance liquid chromatography, maxillofacial levodopa delivery, minimal clinically important difference, motor examination, movement disorder society sponsored revision of the unified parkinson’s disease rating scale, parkinson’s disease

## Abstract

Background: In advanced Parkinson’s disease (PD), the clinical response to oral levodopa diminishes over time, leading to dyskinesia and unpredictable "off" periods. In PD patients with dementia, those with a Ryle’s tube in situ, or those who decline existing levodopa delivery routes such as intraduodenal, subcutaneous, or oral inhalation, alternative approaches are required. This study evaluates a novel maxillofacial route and platform as a clinically viable alternative to conventional levodopa delivery methods.

Objective: This study evaluates the feasibility and safety of low-dose levodopa administered using a maxillofacial platform in a PD patient and assesses systemic exposure and its relationship to clinically meaningful motor outcomes over a 24-hour period following dosing at regular intervals.

Methods: A PD patient received four cycles of 5 mg levodopa (one-twentieth of the patient’s usual oral dose) through a maxillofacial route at regular 3.5-hour intervals. Levodopa was administered using a maxillofacial platform designed to enable controlled, repeated dosing through this route. The patient was monitored over a 24-hour period. Motor outcomes were assessed using the Movement Disorders Society-sponsored revision of the Unified Parkinson’s Disease Rating Scale (MDS-UPDRS), Part III: Motor Examination (ME). Clinically meaningful improvement was evaluated using Minimal Clinically Important Difference (MCID) thresholds. Plasma levodopa concentrations were quantified using high-performance liquid chromatography (HPLC). Local and systemic adverse events were monitored throughout the study period.

Results: MCID-level improvement was observed in 16 (94%) post-dose MDS-UPDRS assessments. Robust improvement was observed in 13 (76%) post-dose assessments. The median improvement from baseline was nine points, the interquartile range (IQR) was eight points (5-13), and the observed range was fourteen points (2-16). Clinically meaningful benefit was detected within 30-60 minutes, peaked at 1-2 hours, and persisted for approximately 3 hours per dose. Robust motor responses occurred across a wide range of systemic exposures, demonstrating a temporal and quantitative dissociation between plasma levodopa area under the concentration-time curve (AUC) and clinical effect. No local or systemic adverse events were observed.

Conclusion: Low-dose levodopa administered at regular intervals through the maxillofacial platform yielded reproducible and clinically meaningful improvement. The platform was safe, effective, and well tolerated over the 24-hour study period.

## Introduction

Oral levodopa remains the gold standard for PD. However, as the disease progresses, the clinical efficacy of oral levodopa reduces because of erratic gastric emptying, competition with dietary amino acids, delayed gastrointestinal absorption, and extensive peripheral metabolism. These factors lead to fluctuating plasma levels and contribute to motor complications, including peak-dose and biphasic dyskinesias. Several alternative levodopa delivery strategies have been developed to address these challenges [1]. However, all existing drug delivery routes have limitations, highlighting the need for an alternative route that can be used in challenging patients, such as those with cognitive impairment, patients with a Ryle’s tube in situ, patients who refuse existing drug delivery routes, and individuals experiencing early-morning "offs," sudden "offs," or dyskinesias.

Nonoral routes of levodopa delivery include continuous small intestinal administration, oral inhalation, and continuous subcutaneous infusion [2-4]. In advanced PD with severe motor fluctuations, continuous small-intestinal infusion of levodopa-carbidopa gel has been used to bypass the stomach. Although the bioavailability is similar to the oral route, a faster and more stable plasma concentration can be achieved compared with oral dosing. It decreases OFF time and increases ON time. However, it requires invasive percutaneous gastrojejunostomy and is associated with device- and procedure-related complications [5, 6]. Oral inhalation bypasses the gut and delivers levodopa into the lungs for rapid absorption into systemic circulation. It has been approved only for intermittent treatment of OFF periods in patients with optimized baseline levodopa therapy. Improvement in MDS-UPDRS-III (Movement Disorders Society-sponsored revision of the Unified Parkinson’s Disease Rating Scale, Part III) score was noted at 10 minutes and was sustained until 60 minutes. Although inhaled levodopa provides rapid symptomatic relief during off periods, it is limited by its short duration of action and does not address baseline motor fluctuations. Cough was the most common adverse event. It is not recommended for patients with asthma or other chronic lung diseases [7-9].

Recently, continuous subcutaneous infusion of the soluble prodrugs foslevodopa and foscarbidopa has been introduced as a less invasive alternative to the intraduodenal or jejunal route. Clinical trials have demonstrated that this approach can achieve continuous, stable systemic levodopa exposure and reduce OFF time. However, it still requires continuous infusion through an external device. Complications include painful nodules and infusion site reactions [10-12].

As the above-described routes of levodopa administration have several disadvantages, there is a need for an alternative route that can provide a clinically meaningful benefit without reliance on continuous infusion. In this context, the maxillofacial route of administration could be useful.

Preclinical studies have shown that maxillofacial administration can successfully deliver drugs into the brain through perineural, paravascular, glymphatic, vascular, and lymphatic pathways [13-15]. Furthermore, our first-in-human translational report compared oral and maxillofacial routes following administration of a single dose of levodopa and provided preliminary evidence of the clinical efficacy of maxillofacial administration of levodopa [16, 17]. Recently, a randomized controlled pilot study in humans demonstrated rapid systemic delivery of a very low dose of metronidazole through the maxillofacial route [18].

This study evaluates the feasibility and safety of low-dose levodopa administered at regular 3.5-hour intervals through a novel maxillofacial drug delivery route and platform and assesses systemic exposure and clinically meaningful motor outcomes over a 24-hour period in a PD patient. It further evaluates the temporal and quantitative relationship between plasma levodopa concentration and the clinical motor response.

A part of this article was previously presented as a meeting abstract at the 2024 International Congress of Parkinson’s Disease and Movement Disorders Meeting on September 27-October 1, 2024, Philadelphia, USA.

## Materials and methods

Study procedure

This study was approved by the Institutional Ethics Committee of Indira Gandhi Government General Hospital and Post Graduate Institute, Puducherry, India. An elderly male with Parkinson’s disease attending the Movement Disorders Clinic in the Department of Neurology was enrolled in the study. The patient fulfilled the UK Brain Bank diagnostic criteria for Parkinson’s disease and was on oral levodopa/carbidopa therapy.

The patient required endodontic treatment for an upper premolar. The pulp cavity was debrided and enlarged. The access opening to the pulp cavity was temporarily closed with dental cement.

The patient fasted overnight, and the morning dose of levodopa/carbidopa was withheld to ensure a washout period of at least 7.5 hours. A predose blood sample was collected. A maxillofacial drug delivery system was temporarily attached to the tooth for drug administration. One-twentieth of the patient’s usual oral levodopa dose (100 mg) was administered through the maxillofacial route using this system. Accordingly, a 1 mL solution containing 5 mg of levodopa was administered over ten minutes during the first cycle. As the patient tolerated the first cycle well, the administration rate was increased to deliver 5 mg over five minutes during each of the remaining three cycles. The maxillofacial drug delivery system was removed after completion of each drug administration cycle. Levodopa was administered in four cycles at regular intervals of 3.5 hours, and the patient was monitored over a 24-hour period. MDS-UPDRS Part III ME scores were assessed by the same examiner at 30, 60, 90, 120, 150, and 180 minutes after each dose. MCID values were then calculated.

During each of the four dosing cycles, post-dose blood samples were collected at 30, 60, 90, 120, 150, and 180 minutes. Plasma was separated by centrifugation, stored at −80°C, and analyzed subsequently. Plasma concentrations of levodopa were quantified using a validated HPLC method with ultraviolet detection at 280 nm (20 µL injection; six-minute run time) on a C18 column under isocratic conditions, using an aqueous buffer-acetonitrile mobile phase. Prior to HPLC analysis, proteins in plasma samples were precipitated using acetonitrile, and the resulting supernatant was analyzed. The AUC was calculated to assess systemic exposure.

Statistical analysis

In this study, multiple analyses were conducted to evaluate clinical response, the quantitative exposure-response association, and temporal alignment between AUC and post-dose motor improvement, expressed as MCID based on the MDS-UPDRS Part III.

Changes from baseline MDS-UPDRS Part III were summarized descriptively using nonparametric methods. Clinically meaningful improvement was classified according to established MCID thresholds.

For quantitative exposure-response assessment, the Spearman rank correlation coefficient (ρ) was used as the primary analysis because of its robustness to nonlinearity and non-normal distributions and was applied to assess the monotonic association between plasma levodopa AUC and motor improvement. Two-sided p-values were calculated, with statistical significance defined as α = 0.05. For comparison, Pearson correlation (r) was used as the sensitivity analysis to evaluate linear association, and simple linear regression was performed to estimate the proportion of explained variance (R²).

Nonlinear exposure-response relationships were explored using logarithmic transformation, Emax modeling, and segmented (piecewise) regression to assess potential saturation or threshold effects.

Temporal alignment was evaluated using forward lag analyses (lag 0, +1, +2) with Spearman correlation as the primary method. Cross-correlation function analysis was performed as a sensitivity analysis to examine potential distributional delay.

IBM SPSS Statistics for Windows, Version 29 (Released 2022; IBM Corp., Armonk, New York) was used for statistical analysis. The statistical significance level was set at α = 0.050. Data visualization was performed using the Matplotlib module in Python 3.

## Results

The pre-dose baseline MDS-UPDRS score was 29. Changes from baseline MDS-UPDRS scores were descriptively summarized using nonparametric methods.

In order to assess whether the observed improvements were clinically meaningful, previously validated MCID thresholds (≥ 3-5 points) for MDS-UPDRS were used. MCID represents the smallest change in a measurement that is clinically meaningful to patients. Post-dose changes of (<3 points) from baseline were classified as below MCID, changes of (≥3-5 points) were classified as minimal MCID, and changes (≥6 points) were classified as robust MCID.

MCID-level improvement was observed in 16 (94%) of post-dose assessments, while robust responses (≥6 points) were observed in 13 (76%) post-dose assessments. Minimal responses (≥3-5 points) occurred at three time points, primarily near the end of dosing intervals and at the next morning assessment, consistent with wearing-off of plasma levodopa concentration. A single below-MCID (< 3 points) observation occurred at the earliest post-dose time point, prior to full onset of effect (Table 1). A median improvement of nine points from baseline, with an interquartile range (IQR) of eight points (5-13), and a range of fourteen points (2-16), was observed. The minimum observed improvement of two points occurred at the earliest post-dose assessment (Table 2). 

**Table 1:** Timeline of drug delivery with corresponding MDS-UPDRS scores, AUC values, and level of MCID MDS-UPDRS: Movement Disorder Society Unified Parkinson’s Disease Rating Scale, AUC: area under the curve, MCID: minimal clinically important difference.

Stage	Time	MDS-UPDRS	AUC	Improvement from baseline	MCID category
Pre-dose baseline	9:00 AM	29	1,204,509	0	Below MCID
Drug delivery 9:45 AM					
Observation	10:15 AM	27	2,142,588	2	Below MCID
Observation	10:45 AM	20	1,940,932	9	Robust MCID
Observation	11:45 AM	14	3,040,212	15	Robust MCID
Observation	12:45 PM	21	2,881,990	8	Robust MCID
Drug delivery 1:15 PM					
Observation	1:45 PM	18	2,330,518	11	Robust MCID
Observation	2:15 PM	16	2,175,675	13	Robust MCID
Observation	3:15 PM	13	2,460,849	16	Robust MCID
Observation	4:15 PM	25	2,616,554	4	Minimal MCID
Drug delivery 4:45 PM					
Observation	5:15 PM	19	2,185,198	10	Robust MCID
Observation	5:45 PM	17	1,678,484	12	Robust MCID
Observation	6:45 PM	21	2,434,387	8	Robust MCID
Observation	7:45 PM	25	2,269,330	4	Minimal MCID
Drug delivery 8:15 PM					
Observation	8:45 PM	21	1,934,399	8	Robust MCID
Observation	9:15 PM	16	2,517,773	13	Robust MCID
Observation	10:15 PM	13	2,618,955	16	Robust MCID
Observation	11:15 PM	23	1,787,808	6	Robust MCID
Observation	8:00 AM (next day)	25	2,225,864	4	Minimal MCID

**Table 2:** Statistics summary

Statistics	Value
Median change from baseline	9 points
Minimum	2 points
Maximum	16 points
Range	14 points (16-2)
Interquartile Range (IQR) (Q3-Q1)	8 points (13–5)
Number of post-dose observations	17
Number of observations ≥ MCID	16/17 (94%)
Number of observations achieving robust MCID	13/17 (76%)
Cumulative time with ≥ MCID response	Approximately 21 hours 15 minutes
Cumulative time with robust MCID response	Approximately 19 hours 15 minutes
Time to onset of MCID response	30–60 min
Duration of robust MCID response	30 min to 3 hours per dose

**Figure 1:**
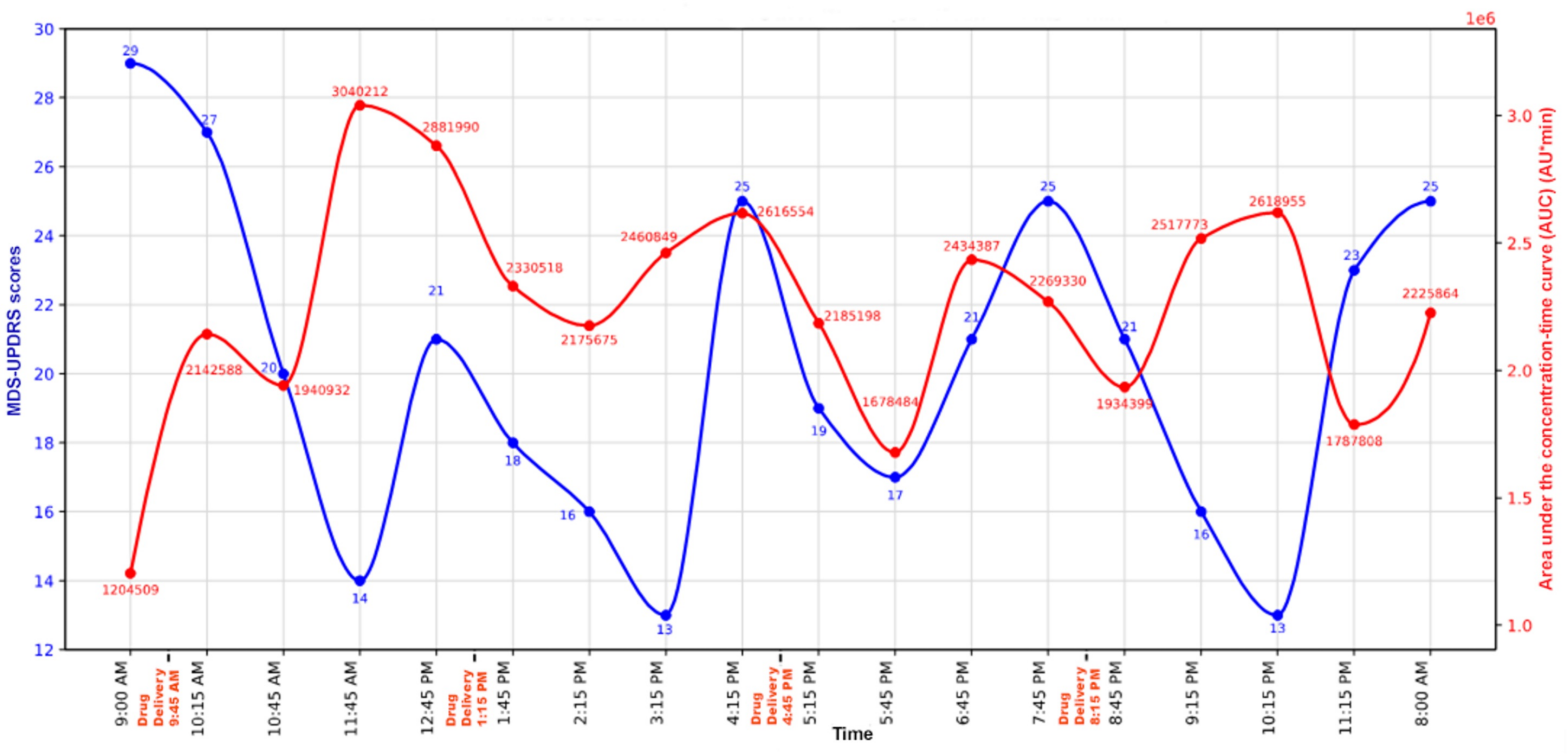
Temporal dissociation between MDS-UPDRS and AUC MDS-UPDRS: Movement Disorder Society Unified Parkinson’s Disease Rating Scale, AUC: area under the curve.

**Figure 2:**
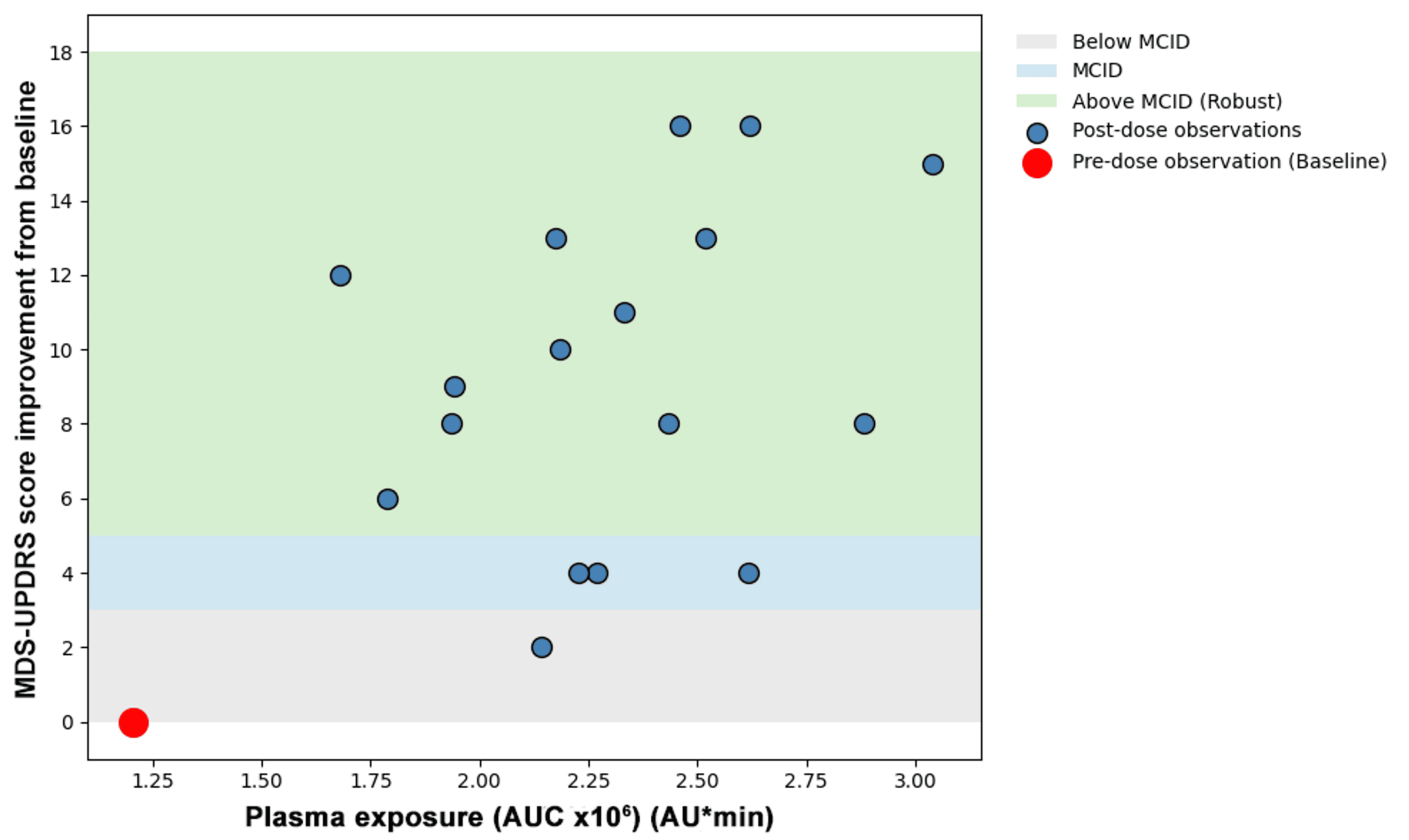
Exposure–response relationship between AUC and MDS-UPDRS motor improvement, stratified by MCID thresholds AUC: area under the curve, MDS-UPDRS: Movement Disorder Society Unified Parkinson’s Disease Rating Scale, MCID: minimal clinically important difference.

After levodopa administration, MCID-level improvements were observed prior to, at, and after the AUC peak time points, indicating clinically meaningful benefit regardless of the exact peak AUC values. Temporal profiling demonstrated a consistent response pattern following each administration. MCID values were reached by 30-60 minutes post-dose, peak improvement was typically observed one to two hours post-dose, and clinically meaningful benefit was maintained for approximately three hours per dose (Figure 1). The distribution of improvements was skewed toward clinically robust responses, with the majority of observations exceeding robust MCID thresholds. Exploratory quantitative exposure-response analysis using scatterplot assessment of plasma levodopa AUC versus change in MDS-UPDRS scores demonstrated substantial vertical dispersion across similar exposure values. Comparable AUC values (approximately 2.0-2.6 × 10⁶ AUmin) were associated with a wide range of clinical motor improvements (2-16 points) (Figure 2).

**Figure 3:**
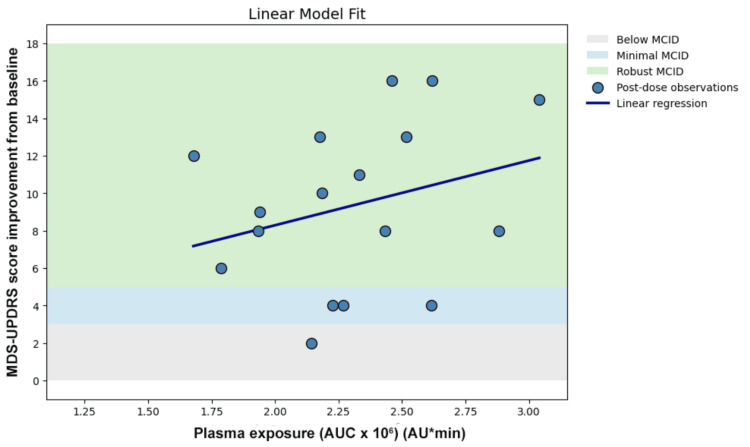
Linear model

**Figure 4:**
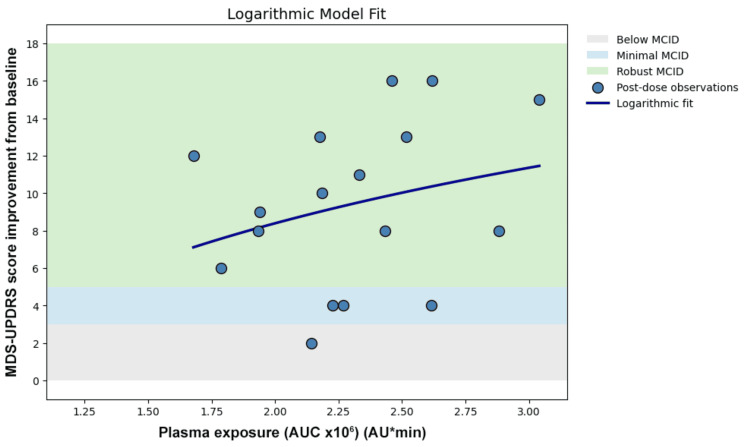
Logarithmic model

**Figure 5:**
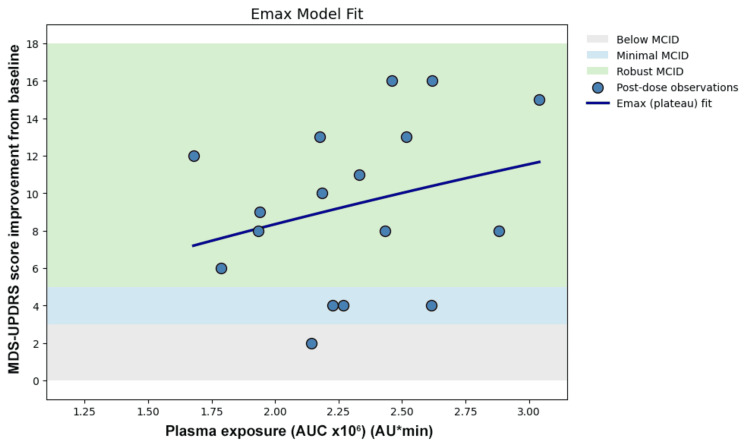
Emax model

**Figure 6:**
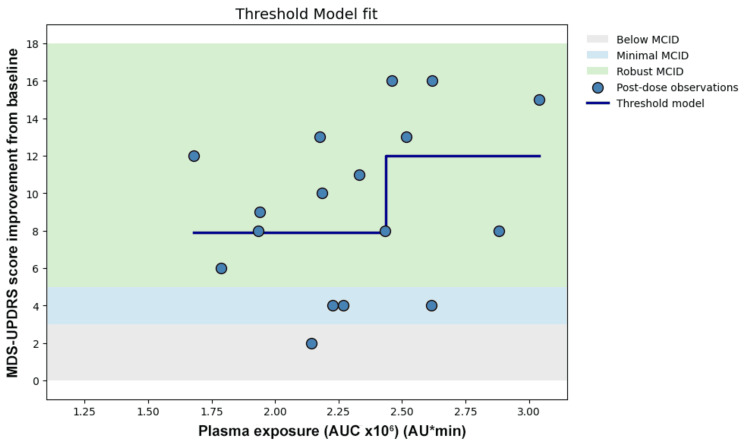
Threshold model

Linear regression demonstrated low explained variance (Figure 3). Logarithmic transformation did not improve the model fit (Figure 4). Nonlinear Emax modeling did not demonstrate plateau behavior within the observed exposure range and did not improve the model fit (Figure 5). Piecewise modeling was performed to evaluate a potential exposure threshold. Although a breakpoint was identified within the exposure range, substantial variability in motor improvement was observed both below and above the estimated threshold. The threshold model did not reduce dispersion or improve the overall model fit (Figure 6). Overall, no meaningful exposure-response relationship was observed within the studied exposure range.

Spearman correlation demonstrated a weak monotonic association between AUC and motor improvement (ρ = 0.31; p > 0.05). Pearson correlation similarly demonstrated a weak linear association (r = 0.29; p > 0.05), with R² = 0.08, indicating that systemic exposure accounted for approximately 8% of the variability in motor improvement (Table 3). 

**Table 3:** Correlation between plasma levodopa AUC and motor improvement Both Spearman and Pearson analyses demonstrated weak and non-significant associations, indicating that systemic exposure accounted for only a small proportion of the variability in clinical response.

Analysis method	Correlation coefficient	p-value	Explained variance	Interpretation
Spearman rank correlation (ρ)	0.31	0.225	—	Weak monotonic association; not statistically significant
Pearson correlation (r)	0.29	0.266	8% (R² = 0.08)	Weak linear association; not statistically significant

**Figure 7:**
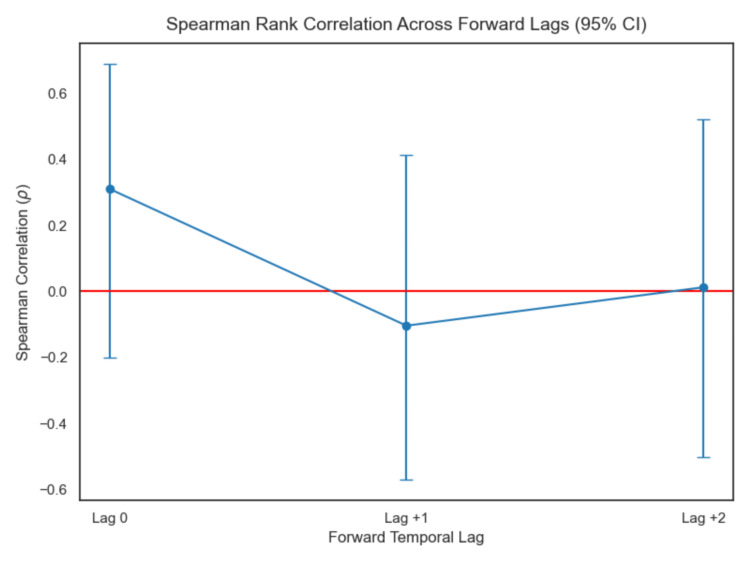
Lag analysis

**Figure 8:**
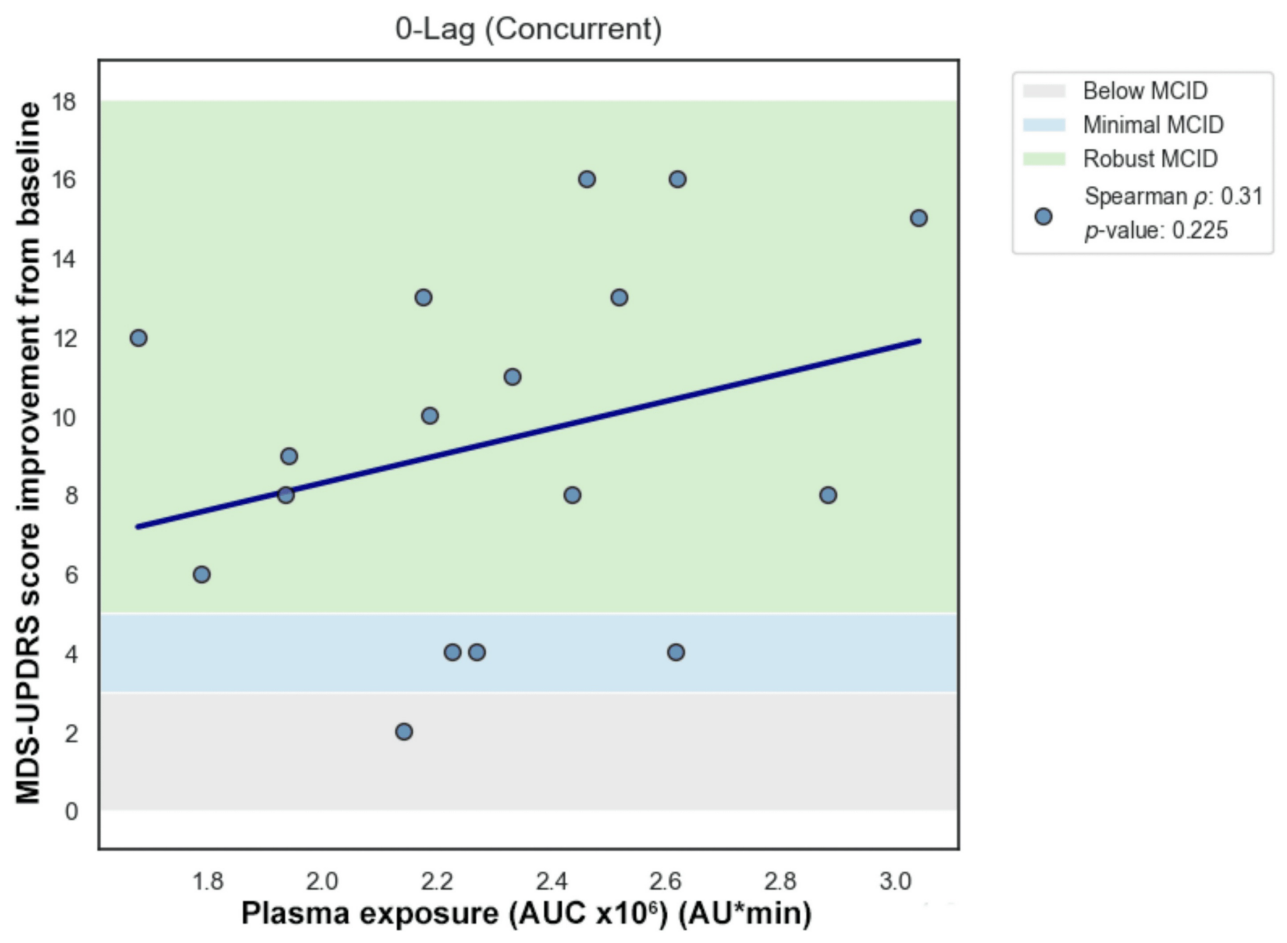
Lag 0

**Figure 9:**
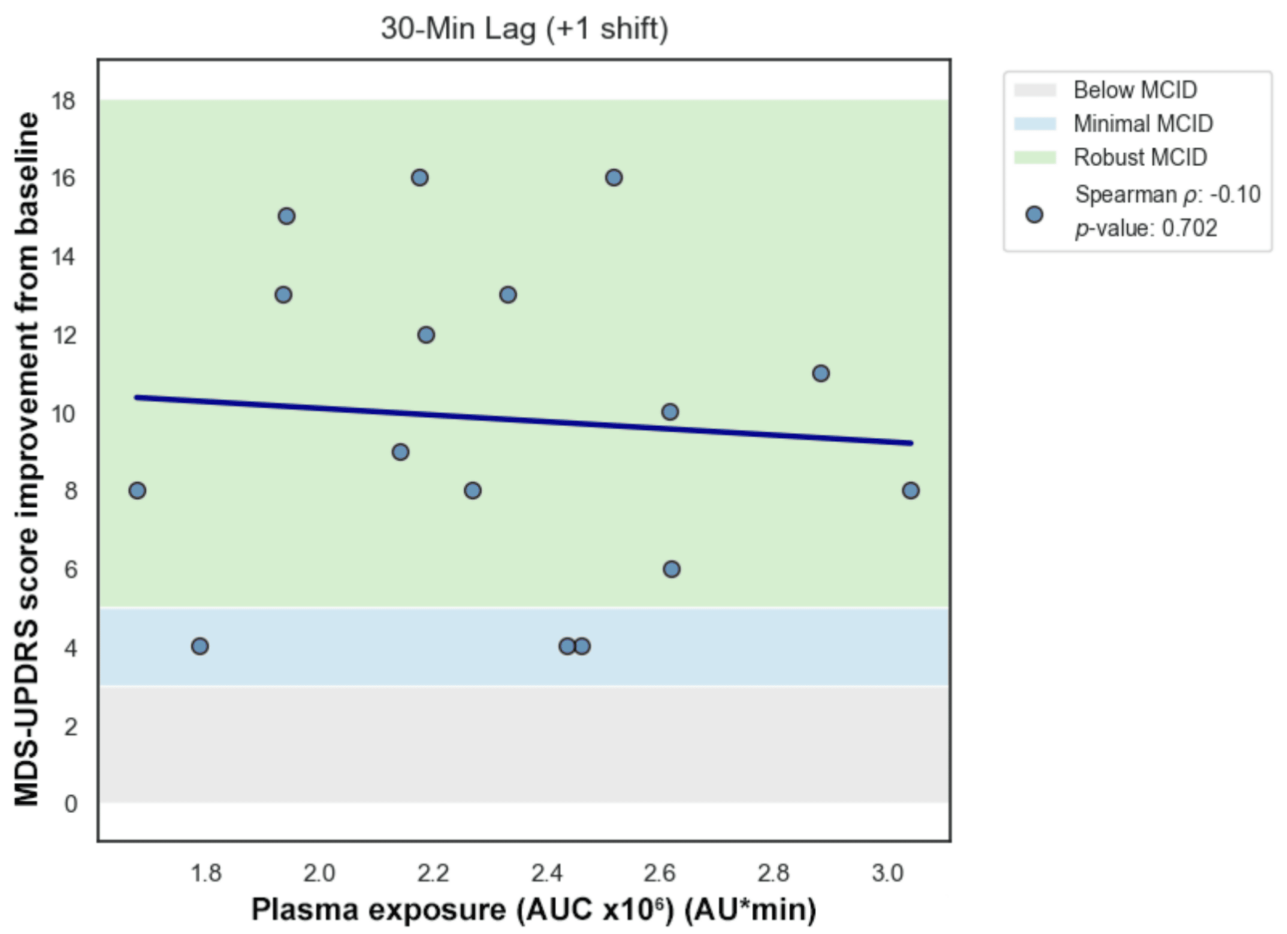
Lag 1

**Figure 10:**
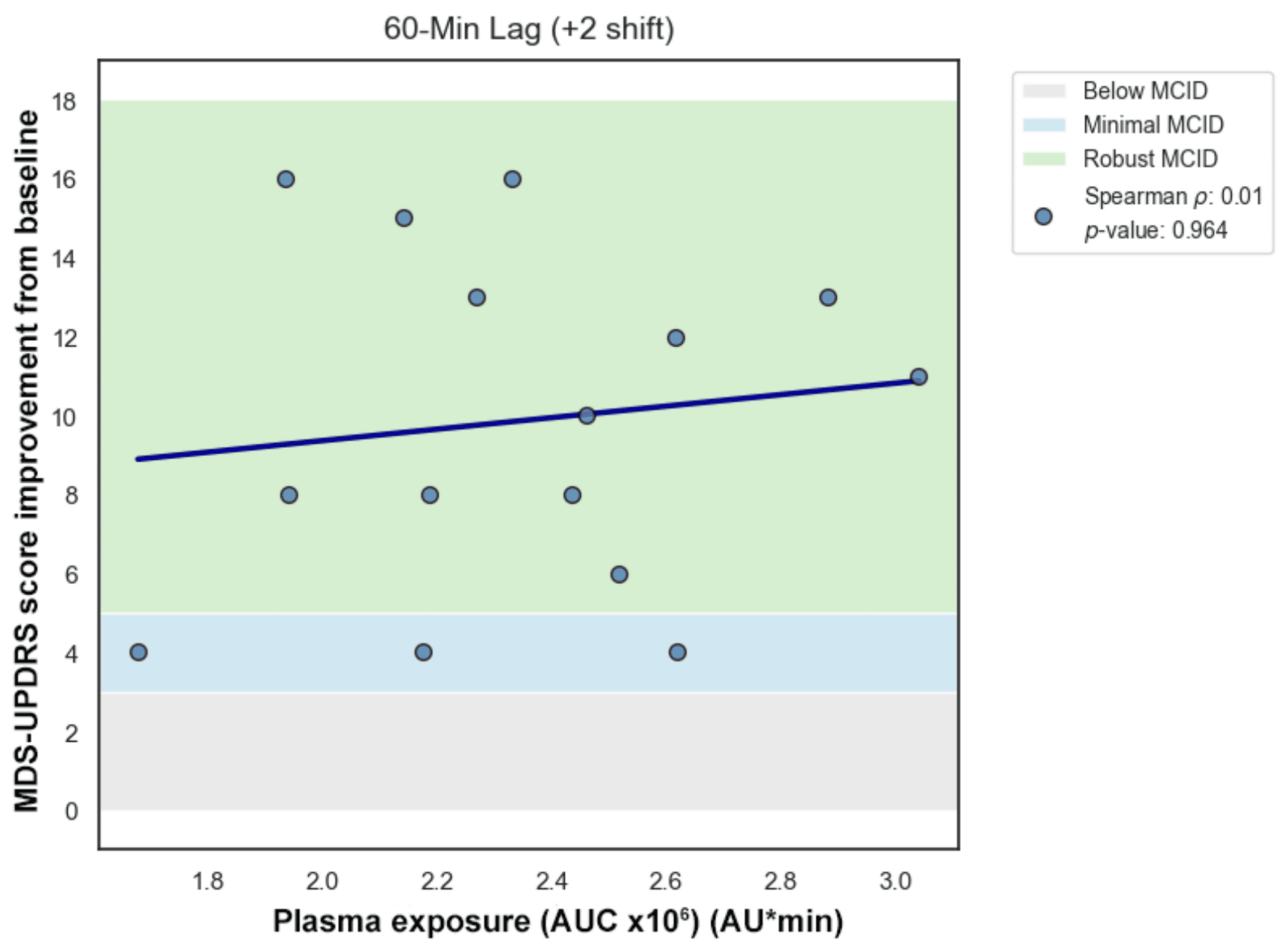
Lag 2

**Figure 11:**
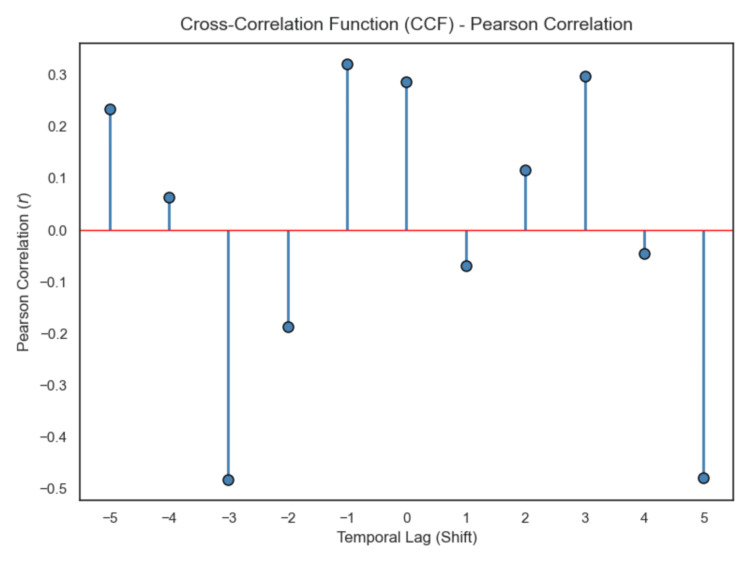
Cross-correlation function analysis

Primary temporal lag analysis was performed manually using Spearman rank correlation across forward lags (lag 0, +1, +2) (Figures 7-10). Correlation coefficients were weak and not statistically significant across the tested lags. Sensitivity analysis using cross-correlation function analysis similarly did not demonstrate a dominant positive lag associated with improved correlation strength (Figure 11). Forward lag adjustment did not meaningfully reduce exposure-response dispersion or restore a monotonic association. 

## Discussion

In this 24-hour pilot study, low-dose levodopa administration through a novel maxillofacial platform at regular 3.5-hour intervals produced rapid, reproducible, and clinically meaningful motor improvement in a patient with PD. Using established MCID thresholds [19, 20] for MDS-UPDRS Part III ME, clinically relevant improvement was observed in the majority of post-dose assessments, with robust responses occurring consistently across four dosing cycles. Importantly, these motor benefits were achieved despite minimal, variable, and nonsustained systemic levodopa AUC. These findings suggest that maxillofacial administration of levodopa may provide symptomatic benefit in PD without reliance on peak or sustained systemic levodopa exposure.

The primary novelty of this study lies in the maxillofacial drug delivery platform and the route of administering levodopa. Existing levodopa delivery routes, such as oral formulations, intestinal gel infusion, and subcutaneous infusion, aim to optimize and stabilize plasma levodopa concentrations through continuous delivery of substantial doses. In contrast, the maxillofacial platform represents a fundamentally different approach: a nongastrointestinal, non-continuous infusion-based system that enables controlled, on-demand administration through the maxillofacial route. To our knowledge, this is the first 24-hour clinical observation in a PD patient to demonstrate that levodopa delivered at regular intervals through the maxillofacial route can produce rapid and clinically meaningful motor benefit. These findings may expand the therapeutic landscape beyond systemic exposure-centric strategies.

A second novel aspect of this study is the low-dose administration enabled by the maxillofacial drug delivery platform. Existing infusion platforms primarily aim to stabilize systemic levodopa levels through continuous infusion of substantial doses delivered using external devices that are secured to PD patients for several hours daily, approximately 16 hours with Duodopa and up to 24 hours with foslevodopa/foscarbidopa. In contrast, the maxillofacial platform required attachment to the patient for only five to ten minutes per administration. Importantly, robust and reproducible MCID-level motor improvements were achieved with administration of very low doses of levodopa at regular intervals through the maxillofacial route, despite the absence of sustained systemic levodopa exposure.

Clinically meaningful motor responses were observed across a wide range of low systemic AUC values, indicating that effective symptom control may be possible without high systemic exposure when levodopa is delivered using the maxillofacial platform. The rapid motor response showed maximal benefit at approximately one to two hours post-dose and persisted for approximately three hours before wearing off. Re-administration consistently restored robust clinical benefit, supporting a direct pharmacologic effect.

From a clinical perspective, the observed response profile may have therapeutic implications. The relatively rapid onset and reproducible restoration of benefit may be particularly relevant for managing early-morning "off" periods, sudden "off" episodes, or patients unable to tolerate or access existing infusion-based systems. The absence of sustained systemic exposure within the observed range also raises the possibility that this approach may mitigate peak-dose-related adverse effects. Reproducible clinical responses across four dosing cycles within the same patient strengthen the internal validity of these observations despite the exploratory nature of the study. Notably, attenuation of clinical benefit to minimal MCID levels by the following morning supports a treatment-dependent effect with limited overnight durability, rather than a nonspecific or placebo-driven response.

In Parkinson’s disease, levodopa exposure-response relationships are traditionally conceptualized as monotonic once effect-site equilibration is considered. Plasma concentration (AUC) is generally expected to predict motor improvement after accounting for blood-brain barrier transport and central dopaminergic processing. At lower exposure ranges, this relationship is often near-linear. At higher ranges, it typically demonstrates saturable (Emax-type) behavior. Temporal hysteresis is also well described, but alignment for distributional delay ordinarily restores an orderly exposure-response pattern. Dissociations between plasma levodopa concentrations and clinical motor response have been previously described with conventional delivery routes and are therefore not novel in themselves [21-25]. However, the present study demonstrates a reproducible dissociation between plasma levodopa concentration and clinical motor response within the same individual despite the low dose.

Two complementary forms of dissociation were observed. First, a quantitative dissociation was identified. In the present dataset, monotonic levodopa exposure-responses were not consistently observed across the four dosing cycles. Comparable AUC values were associated with markedly different MCID levels of motor improvement, and higher exposure did not reliably correspond to greater clinical benefit. Linear regression demonstrated substantial dispersion (Figure 3), and nonlinear approaches such as logarithmic transformation (Figure 4), Emax modeling (Figure 5), and threshold modeling (Figure 6) did not improve the model fit within the observed exposure range. Figure 2 illustrates the quantitative dissociation between AUC and clinical motor response, demonstrating that similar AUC values (2.0-2.6 × 10⁶) produced MCID improvements ranging from 2 to 16 points in the same patient. If systemic exposure were the sole determinant of efficacy, MDS-UPDRS scores should scale monotonically with increasing AUC, with minimal vertical dispersion. On the contrary, substantial vertical dispersion of MCID scores is seen at similar AUC values. Most of these observations show robust scores without proportionally higher AUC exposure. These findings suggest that systemic AUC values may not be the sole contributing factor for the observed clinical improvement following maxillofacial administration.

Second, a temporal dissociation was evident, with clinically meaningful motor improvement frequently occurring before, coinciding with, or persisting beyond plasma AUC peaks. Figure 1 shows the temporal dissociation between AUC and clinical motor response. From a pre-dose baseline MDS-UPDRS score of 29, clinically relevant improvement was observed within 30-60 minutes of dosing, with robust improvements sustained at most post-dose time points. The AUC at 10:15 AM was 2.14 × 10⁶ AUmin with an improvement of only 2 points, below MCID. Although the AUC dropped to 1.94 × 10⁶ AUmin at 10:45 AM, there was a nine-point robust MCID improvement from baseline. In the later part of the day, lower AUC values produced robust improvement, whereas higher AUC values produced minimal improvement at several time points, for example, 3:15 PM versus 4:15 PM. Across all four dosing cycles, a reproducible pattern of rapid improvement, sustained benefit, and lack of alignment with AUC peaks was observed. There was a clear temporal dissociation between clinical motor responses and AUC peaks, as robust clinical improvement frequently occurred independent of AUC peaks at multiple time points, as shown in Table 1 and Figure 1.

Because temporal misalignment can create apparent dissociation, exploratory lag-adjusted analyses were performed by forward-shifting exposure values across sampling intervals (Figures 7-11). If classical effect-site delay were the primary explanation, such alignment would be expected to strengthen correlation and reduce dispersion. However, lag adjustment did not meaningfully restore a monotonic relationship. Importantly, while temporal shifting can alter alignment, it does not generate vertical dispersion at similar exposure levels, suggesting that plasma concentration alone does not account for the observed variability.

The reproducibility of both quantitative and temporal dissociation across repeated dosing cycles suggests that part of the observed effect may be driven by mechanisms other than plasma AUC. Meaningful motor benefit was not confined to peak AUC levels, suggesting that clinical response may be influenced by factors beyond systemic exposure alone. These observations raise the possibility that alternative pathways associated with the maxillofacial route may contribute to the therapeutic effect. However, the plausibility of such pathways should be interpreted cautiously because tracers or imaging were not used in the present study. Nonetheless, the observed concentration-motor response dissociation appears consistent with mechanisms extending beyond plasma levodopa pharmacokinetics.

There are limitations to this pilot study. Inter-individual variability, long-term safety with repeated use, and effects in patients with dyskinesia were not evaluated. Additionally, the exploratory analyses were limited by the sample size. Despite these limitations, the consistent and clinically meaningful motor responses observed at low systemic exposure provide a strong rationale to investigate whether low-dose maxillofacial levodopa administered at regular intervals can produce rapid symptomatic benefit in PD. Larger controlled studies are required to confirm reproducibility, assess exposure-response relationships, and define the clinical role of the maxillofacial delivery platform within the therapeutic landscape of Parkinson’s disease.

## Conclusions

The maxillofacial route and platform for levodopa administration were safe, effective, well tolerated, and associated with rapid, reproducible, and clinically meaningful motor improvement in a patient with PD. If validated in larger studies, this novel approach may offer a minimally invasive option for managing early-morning "offs," sudden "offs," dyskinesias, and patients who are unable to tolerate or do not have access to existing nonoral levodopa delivery systems.
